# Editorial: Integrating age structural change into global policy

**DOI:** 10.3389/fpubh.2022.1005907

**Published:** 2022-10-20

**Authors:** Sarah Harper, Kenneth Howse, Angelique Chan

**Affiliations:** ^1^Oxford Institute of Population Ageing, University of Oxford, Oxford, United Kingdom; ^2^Centre for Ageing Research & Education, Duke-NUS Medical School, Singapore, Singapore

**Keywords:** age structural change, policy, economics, care, society

As the UN 2022 Population projections report (1), the population above age 65 years is growing more rapidly than the population below that age. As a result, the share of global population at ages 65 and above is projected to rise from 10 per cent in 2022 to 16 per cent in 2050. At that point, it is expected that the number of persons aged 65 years or over worldwide will be more than twice the number of children under age 5 and about the same as the number under age 12. Measures are thus required to adapt public programmes to the growing numbers of older persons through, for example, the establishment of universal health care and long-term care systems and by improving the sustainability of social security and pension systems. We, as the papers in this volume demonstrate, argue that a far more radical approach is required—one that integrates age structural change into national and global policies.

Most High Income Countries^1^ (HICs) have aged continuously over the past century, using the measure of aging: an increase in the percentage of those over 60 years, and a decrease in those under 15 years. By 2030 half the population of Western Europe will be aged over 50, with a predicted average life expectancy at age 50 of a further 40 years, that is half Western Europe's population will be between 50 and 100 years of age. One quarter of this population will be over 65, 15 per cent over 75. Already two-thirds of the world's older population live in Lower and Middle Income Countries (LMICs), with the absolute numbers of older people in these regions estimated to double to reach some 900 million within 25 years.

As Harper (2) has discussed elsewhere, we need to separate the drivers behind such demographic change—that is the declining numbers of children being born combined with the lengthening of life of those already born. There are clearly separate though linked dimensions to this demographic future, which need to be considered. Falling^1^ fertility leads to *age structural change*—the shift in population ages from predominantly young to predominantly older populations. Such a shift in the age structure of a population has clear implications for the distribution and allocation of societal resources between age groups and generations.

Falling mortality leads to increases in life expectancy initially at birth, and then at all ages, while falling late life-mortality also results in the *growth in the number and proportion of oldest old*. This latter trend in particular increase demands for the societal resources to support a potential lengthening of dependency and frailty at the end of our lives. This has implications for financial, health and social care support.

While Harper does not argue that longevity increase and age-structural change have independent influences, it is recognized that longevity has a greater impact on the increased need for financial and care support for these longer lives. Age structural change will have a greater impact on the structure within which our lives are lived such as work, education, housing and families.

It is on the generational economy that the contribution by Rice et al. focus. Discussing the economic activities of, and relationships between, different ages and generations, they highlight the critical role of the financial sustainability of the generational economy, the intergenerational inequality that the generational economy creates, and the associated material living standards. Taking the case example of Australia, they consider how the generational economy can be expected to perform in the coming decades in terms of financial sustainability, intergenerational inequality, and material living standards. Performance is evaluated under a variety of demographic and economic scenarios delineated on the basis of projected changes in fertility, mortality, overseas migration, and labor income growth.

Moving onto health care, Lu and Liu highlight the importance of the growth in the need of palliative care, as the number and proportion of those approaching death will grow within all aging populations. They suggest that the quality of earlier social relationships is a significant predictor of people's awareness of and attitude toward palliative care. Taking a societal approach, they strongly argue that public health campaigns and education aiming at changing people's views of palliative care, must take this into account as these will ultimately improve end-of-life outcomes. Importantly, as Calvo-Sotomayor and Atutxa remind us in their paper, older adults are carers also. Employing the commons paradigm as a framework for understanding the positive contribution of older cohorts to society, they highlight that the care activities of older adults are essential to European Society, thus reversing the preconception that older people are a negative drain, through establishing that they are an essential part of the common good. This theme is extended by Moon with a cost prediction of an integrated care system in Korea, based on a needs based approach, arguing that important factors for the success of community care are beneficiary satisfaction and sustainable financial operations.

The volume is completed with the bibliometric study Exploring the Relationships Between Four Aging Ideals: A Bibliometric Study by Lin et al. which identifies 4 complementary aging ideals: successful aging, healthy aging, productive aging and active aging.

As the papers in this volume demonstrate, age-structural change must be integrated into our understanding of human, societal and economic development, Policy frameworks need to be adjusted, and indeed already are being adjusted, to reflect the upcoming demographic shift. Acknowledging the importance of age-structural change, and ensuring that it is integrated in national and international policymaking will be essential as the globe transitions from a predominantly young to a predominantly older world.

## Author contributions

All authors listed have made a substantial, direct, and intellectual contribution to the work and approved it for publication.

## Conflict of interest

The authors declare that the research was conducted in the absence of any commercial or financial relationships that could be construed as a potential conflict of interest.

## Publisher's note

All claims expressed in this article are solely those of the authors and do not necessarily represent those of their affiliated organizations, or those of the publisher, the editors and the reviewers. Any product that may be evaluated in this article, or claim that may be made by its manufacturer, is not guaranteed or endorsed by the publisher.

## Footnotes

^1^Definition of HICs and LMICs is a World Bank definition: high income countries (HICs) are countries with a GNI per capita above US$ 12,746, medium income countries (MICs)—are countries with a GNI per capita of more than $1,045 but less than $12,746, low income countries (LICs)—are countries with a GNI per capita of $1,045 or less.
